# Characterizing the NIH Activity and Chronicity Indices in 2 Independent Lupus Nephritis Cohorts

**DOI:** 10.1016/j.ekir.2025.11.011

**Published:** 2025-11-20

**Authors:** Valentina Querin, Natasha Jordan, David P. D’Cruz, David Isenberg, Suzanne Wilhelmus, Helmut Schumacher, H. Terence Cook, Augusto Vaglio, Ingeborg M. Bajema

**Affiliations:** 1 Department of Pathology and Medical Biology, University of Groningen, University Medical Center Groningen, Groningen, The Netherlands; 2 Department of Biomedical, Experimental and Clinical Sciences “Mario Serio” University of Florence, Firenze, Italy; 3 Rheumatology Department, St James’s Hospital, Dublin, Ireland; 4 School of Medicine, Trinity College Dublin, Dublin, Ireland; 5 The Louise Coote Lupus Unit, Guys and St Thomas’ Hospitals, London, UK; 6 Division of Medicine, Department of Ageing, Rheumatology and Regenerative Medicine, University College London, London, UK; 7 Pathan B.V., Laboratory for Pathology, Rotterdam, The Netherlands; 8 Statistical Consultant, Ingelheim, Germany; 9 Department of Immunology and Inflammation, Imperial College London, London, UK; 10 Nephrology and Dialysis Unit, Meyer Children’s Hospital IRCCS, Firenze, Italy

**Keywords:** activity index, chronicity index, histopathological classification, lupus nephritis

## Abstract

**Introduction:**

The inclusion of National Institutes of Health (NIH) activity index (AI) and chronicity index (CI) in the ISN/Renal Pathology Society (ISN/RPS) classification of lupus nephritis (LN) aims to provide a precise characterization of the amount of active and chronic lesions next to lupus class. We here investigate the distribution of NIH indices within 2 international LN cohorts, their relationship with the ISN/RPS classes and which lesions most significantly contribute to these scores.

**Methods:**

We collected 194 biopsies from 2 cohorts of patients with LN and calculated the NIH AI and CI according to the revised 2018 ISN/RPS classification. For statistical analysis we mainly used nonparametric tests. An exploratory factor analysis was applied to the lesion scores.

**Results:**

The NIH AI score was usually medium-low, reaching a maximum value of 16 of 24, whereas the NIH CI reached 10 of 12. Both indices were higher in classes III, IV, and mixed compared with others (*P* < 0.0001). Endocapillary hypercellularity was present in > 70% of biopsies, showing a strong correlation with neutrophils/karyorrhexis (*r* = 0.78, *P* < 0.0001) and cellular crescents (*P* < 0.0001). Chronic lesions showed a strong correlation with each other (*P* < 0.0001), except for fibrous crescents which had the strongest correlation with cellular crescents (*r* = 0.33, *P* < 0.0001). The inclusion of all lesions in an exploratory factor analysis uncovered 2 underlying main factors that accurately reflect the NIH AI and CI.

**Conclusion:**

This study revealed key aspects of the NIH AI and CI that may guide future modifications of these indices, leading to a more balanced and reliable scoring system.

## Graphical abstract

**Figure:**
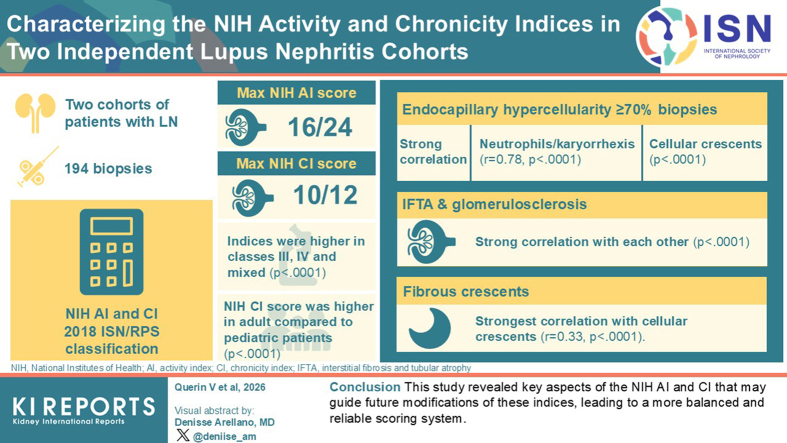

Kidney biopsies of patients with systemic lupus erythematosus exhibit significant heterogeneity, often presenting a complex combination of active and chronic lesions. These lesions can combine to show a variety of patterns which are categorized according to the ISN/RPS classification for LN.^1^ This classification is widely adopted; however, it does not fully capture all the nuances of biopsy findings. Within each class, there can be considerable variability in types of lesions and their more acute or chronic phase for which the NIH AI and CI were proposed by Austin *et al.*^2^^,^^3^ in 1983. Their important role in addition to the determination of lupus class was emphasized in the revision of the ISN/RPS LN classification of 2018.^1^ Usage of the NIH AI and CI is considered to provide a more precise histological characterization, and potentially enhance the prognostic value of kidney biopsies and assist in clinical decision-making. The indices yield a semiquantitative assessment of the overall activity and chronicity detected in renal tissue and are recommended to be applied to all classes of LN.^1^ Unlike the classification, the NIH AI and CI include lesions from the tubulointerstitial compartment that have been demonstrated to play a significant prognostic role, independent from glomerular damage.^4^, ^5^, ^6^

The use of NIH AI and CI in both clinical and research settings is widespread. Their utility includes assessing the appropriateness of immunosuppressive treatment versus conservative management for chronic kidney disease, and predicting patient outcomes. However, several studies following the seminal article by Austin *et al.*^2^^,^^3^ yielded conflicting results regarding the prognostic significance of the NIH AI and CI scores.^7^, ^8^, ^9^, ^10^, ^11^, ^12^, ^13^ Since the 2018 revision that incorporated the NIH indices into the ISN/RPS classification, validation studies have consistently demonstrated that the NIH CI is highly effective in predicting critical outcomes, such as all-cause mortality and progression to end-stage renal disease. In contrast, the NIH AI has been shown to offer only limited prognostic value.^14^, ^15^, ^16^, ^17^, ^18^ Explanations for this discrepancy range from the inherent heterogeneity of active lesions that may respond differently to immunosuppressive treatments, to the lower weight assigned to tubulointerstitial activity in the overall scoring and to the exclusion of vascular lesions. To date, no published studies have critically questioned the behavior of the indices and how they perform in LN cohorts.

All these considerations raise questions about what extent the indices truly reflect the activity and chronicity within the kidney tissue and provide clinically relevant information. The aim of this study was to analyse the distribution of NIH AI and CI within 2 international LN cohorts, to identify the “real world” ranges of the indices, to explore their relationship with the histological ISN/RPS classes, and to evaluate which lesions most significantly contribute to these scores.

## Methods

### Patient Selection

We collected and analysed renal biopsy samples from adult and pediatric (aged ≤ 18 years) patients with LN from 2 international cohorts. All patients fulfilled the 1997 American College of Rheumatology revised criteria for systemic lupus erythematosus.^19^ Cohort 1 included kidney biopsies of 90 patients, primarily from the University Medical Center of Groningen, Groningen, The Netherlands (*n* = 76) and to a lesser extent from the Meyer Children’s Hospital IRCCS in Florence, Italy (*n* = 14). Cohort 2 included kidney biopsies of 104 patients collected from the University College London Hospitals NHS Foundation Trust, UK. Patients from Florence and those included in the UK cohort gave consent for the use of histological data for research purposes. Use of patient kidney biopsies from Groningen was approved by the Central Ethics Review Board non-WMO studies of the University Medical Center of Groningen, registration number 21986. The study was conducted in accordance with the requirements of the Helsinki Declaration.

### Evaluation of Kidney Histopathology

We included native kidney biopsies that were performed at diagnosis, during a disease flare, or to assess histological remission. There was 1 biopsy per patient. Biopsies with < 10 scorable glomeruli or with concomitant glomerular diseases (e.g., diabetic nephropathy, antineutrophil cytoplasmic autoantibody glomerulonephritis) were excluded. An experienced senior nephropathologist (IB) evaluated and scored all the kidney biopsies using light microscopy according to the ISN/RPS 2018 revised classification.^1^ Glomeruli were included in the evaluation if they had ≥ 3 mesangial areas; glomeruli that were either too small or fragmented at the tissue section edge were excluded from assessment.

**Figure 1:**
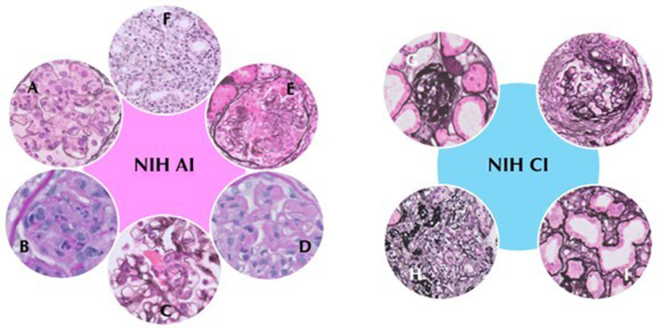
On the left, the 6 lesions included in the NIH AI (0-24) are shown: (a) Endocapillary hypercellularity (0-3), (b) Neutrophils/karyorrhexis (0-3), (c) Fibrinoid necrosis (0-3)x2, (d) Hyaline deposits (0-3), (e) Cellular/fibrocellular crescents (0-3)x2, (f) Interstitial inflammation (0-3). On the right, the 4 lesions included in the NIH CI are shown (0-12): (g) Global glomerulosclerosis (0-3), (h) Interstitial fibrosis (0-3), I. Tubular atrophy (0-3), L. Fibrous crescents (0-3). NIH AI, National Institutes of Health activity index; NIH CI, National Institutes of Health chronicity index.

The NIH AI and CI were determined according to the revised ISN/RPS 2018 classification system.^1^ For the NIH AI, each parameter (endocapillary hypercellularity, neutrophils/karyorrhexis, wire loops/hyaline thrombi, cellular/fibrocellular crescents, fibrinoid necrosis, and interstitial inflammation) was scored from 0 to 3 based on the percentage of affected glomeruli or involved interstitial tissue (< 25%, 1 +; 25%–50%, 2 +; and > 50%, 3 +); following the NIH AI, fibrinoid necrosis and cellular/fibrocellular crescents were assigned double weight. The NIH CI was obtained by scoring total glomerulosclerosis (including segmental and global glomerulosclerosis), fibrous crescents, and tubular atrophy and interstitial fibrosis from 0 to 3 (Figure 1).

### Statistical Analysis

Analyses were performed using SAS statistical software version 9.4 (SAS Institute Inc). Descriptive statistics for continuous variables were reported as median and interquartile range because the data were not normally distributed. The nonparametric Mann-Whitney U test was employed to assess differences in NIH AI and CI scores between groups (cohorts, female vs. male gender, ethnicity, pediatric vs. asdult, and histological classes). Stratified analyses were performed to rule out the influence of cohort-specific differences (adults vs. pediatrics, number of glomeruli) on the observed results. For the analysis of categorical lesion scores the chi-square test was used. Bivariate correlations between active and chronic histopathological lesions were analysed using Spearman’s rank correlation coefficient. For this analysis, the original ordinal scores (0−3) for fibrinoid necrosis and cellular/fibrocellular crescents were used, without duplication. As a sensitivity check, Pearson correlation coefficients were calculated, yielding results similar to the Spearman correlation coefficients. Given the consistent findings across both parametric and nonparametric correlation methods, and acknowledging the robustness of linear statistical methods in case of deviations from the normality assumption, an exploratory factor analysis was performed to investigate whether biopsy lesions could be explained by some underlying latent variables. All 10 individual active and chronic lesion scores were included in this analysis. The number of relevant factors was determined according to the Kaiser criterion, that is, the number of eigenvalues > or close to 1. Following factor extraction and Varimax rotation, the factor loadings were examined to determine the composition of each factor. In this exploratory analysis, various solutions with different numbers of factors were examined and compared. In all statistical analyses, a *P*-value < 0.05 was considered to indicate statistical significance.

## Results

### Study Cohorts

The baseline demographic and histopathological data of patients with LN in both cohorts are shown in Table 1. Specifically, in cohort 1, 72.2% were female; the median age of the patients was 29 years (interquartile range: 16–47.3), with 28 (31.1%) pediatric patients (aged ≤ 18 years). In cohort 2, the proportion of female patients was only slightly higher (81.7%, *P* = 0.11), and there was a lower number of pediatric patients (11.5% vs. 31.1%, *P* = 0.0008). The median number of glomeruli per biopsy was higher in cohort 2 (15 vs. 21, *P* = 0.0002). At stratified comparison, there was no indication that the 2 cohorts substantially differed within the respective strata (adult and pediatric patients; < 17 and ≥ 17 glomeruli) (Supplementary Table S1).

**Table 1:** Demographic and histopathological data of the patients included in the 2 cohorts

Demographic and histopathological data	Cohort 1 (*n* = 90)	Cohort 2 (*n* = 104)	Entire cohort (*N* = 194)	*P*-value
Age, median (Q1–Q3)	29 (16–47)	32 (25–40)	31 (21–42)	0.45
< 18 yrs, *n* (%)	28 (31.1)	12 (11.5)	40 (20.6)	0.0008
Female gender, *n* (%)	65 (72.2)	85 (81.7)	150 (77.3)	0.11
No of glom*,* median (Q1–Q3)	15 (12–20)	21 (13–29)	17 (12–24)	0.0002
Class I, *n* (%)	2 (2.2)	2 (1.9)	4 (2.1)	0.67
Class II, *n* (%)	5 (5.6)	12 (11.5)	17 (8.8)	
Class III, *n* (%)	26 (28.9)	28 (26.9)	54 (27.8)	
Class IV, *n* (%)	39 (43.3)	49 (47.1)	88 (45.4)	
Class V, *n* (%)	10 (11.1)	8 (7.7)	18 (9.3)	
Class III+V, *n* (%)	3 (3.3)	2 (1.9)	5 (2.6)	
Class IV+V, *n* (%)	5 (5.6)	3 (2.9)	8 (4.1)	
Classes III, IV and mixed, *n* (%)	73 (81.1)	82 (78.8)	155 (79.9)	0.69
NIH AI score, median (Q1–Q3)	4 (2–6)	4 (1–8)	4 (1–8)	0.32
Endocapillary hypercellularity, *n* (%)	67 (74.4)	74 (71.2)	141 (72.7)	0.61
Neutrophils/karyorrhexis, *n* (%)	53 (58.9)	60 (57.7)	113 (58.2)	0.87
Fibrinoid necrosis, *n* (%)	12 (13.3)	9 (8.7)	21 (10.8)	0.30
Hyaline deposits, *n* (%)	27 (30)	26 (25)	53 (27.3)	0.44
Cellular/fibrocellular crescents, *n* (%)	31 (34.4)	61 (58.7)	92 (47.4)	0.0008
Interstitial inflammation, *n* (%)	29 (32.2)	44 (42.3)	73 (37.6)	0.15
NIH CI score, median (Q1–Q3)	2 (0–4)	1 (0–4)	2 (0–4)	0.67
Total glomerulosclerosis, *n* (%)	49 (54.4)	61 (58.7)	110 (56.7)	0.56
Fibrous crescents, *n* (%)	13 (14.4)	23 (22.1)	36 (18.6)	0.17
Tubular atrophy, *n* (%)	44 (48.9)	46 (44.2)	90 (46.4)	0.52
Interstitial fibrosis, *n* (%)	47 (52.2)	47 (45.2)	94 (48.5)	0.33

NIH AI, National Institutes of Health activity index; NIH CI, National Institutes of Health chronicity index.

The distribution of the various histological classes was similar in both cohorts, with a higher prevalence of classes III (28.9% and 26.9%) and IV (43.3% and 47.1%) compared with classes II (5.6% and 11.5%), V (11.1% and 7.7%), or mixed (8.9% and 4.8%). The proportion of class I biopsies in the 2 cohorts was 2.2% and 1.9%, respectively.

For cohort 2, we were also able to provide patient ethnicity data, showing a heterogeneous LN population, mostly including Caucasian (38.5%), Afro-Caribbean (37.5%), and Asian patients (20.2%) (Supplementary Table S2).

### NIH AI and CI

The median NIH AI score including both cohorts was 4 (interquartile range: 1–8); the comparison between the cohorts was not significant (*P* = 0.32). There were no significant differences in NIH AI scores according to patient sex (*P* = 0.084) or age (adult vs. pediatric patients, *P* = 0.17), and there was a considerable variation among different ISN/RPS histological classes, because it was significantly higher in classes III, IV, and mixed compared with the others (I, II, V) (median 5 vs. 0, *P* < 0.0001). In cohort 2, we found higher scores for fibrinoid necrosis and hyaline deposits in Asian patients (*P* = 0.039 and *P* = 0.023, respectively), whereas there were no significant differences in the overall NIH AI scores (Supplementary Table S1).

**Figure 2:**
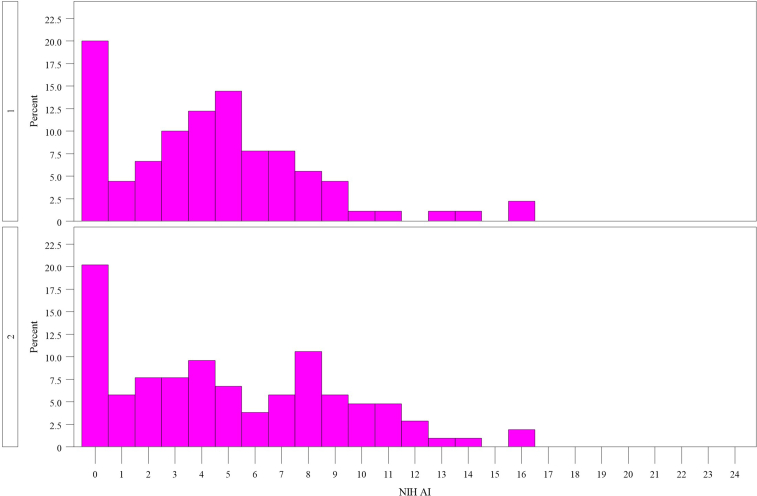
Distribution of the NIH AI score in cohort 1 (above) and cohort 2 (below). NIH AI, National Institutes of Health activity index.

Looking at the distribution of the NIH AI, a noteworthy proportion of biopsies in each cohort exhibited a score of 0, accounting for about 20% in both cohorts (Figure 2). A score of 0 was present across all histological classes; however, it was particularly prevalent in classes I, II, and V compared with classes III and IV. In cohort 1, one-third of biopsies clustered between scores of 3 and 5, whereas cohort 2 showed a more even distribution of NIH AI scores. The highest NIH AI score reached was 16, observed in only 2 biopsies across both cohorts.

**Figure 3:**
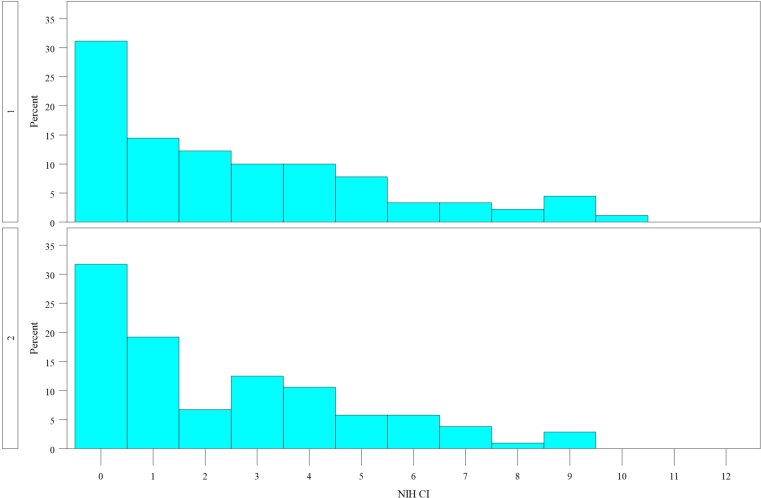
Distribution of the NIH CI score in cohort 1 (above) and cohort 2 (below). NIH CI, National Institutes of Health chronicity index.

When examining the NIH CI, the median score including both cohorts was 2 (interquartile range: 0–4). There were no significant differences in NIH CI scores between the 2 cohorts (*P* = 0.67). The overall distribution of scores was comparable between the 2 cohorts, with about 31% of biopsies in both cohorts scoring 0 (Figure 3). In cohort 1, 92.2% of the biopsies had a score of ≤ 7; whereas in cohort 2, the majority (92.3%) scored ≤ 6. The maximum score observed was 10 in cohort 1 and 9 in cohort 2. There were significantly higher NIH CI scores in patients with classes III, IV, and mixed than in the others (I, II, and V) (median 2 vs. 0, *P* < 0.0001) and in adults than in pediatric patients (median 2 vs. 0, *P* < 0.0001). In addition, the individual scores for total glomerulosclerosis, tubular atrophy, and interstitial fibrosis were higher in adults than in pediatric patients (*P* < 0.005). In cohort 2, there were no significant differences in the NIH CI and chronic lesion scores according to ethnicity.

### Active and Chronic Lesions: Frequency and Scores Distribution

In Table 1, we illustrate that the prevalence of active and chronic lesions in the kidney biopsies of the 2 cohorts was nearly comparable, except for cellular/fibrocellular crescents which were significantly more frequent in cohort 2 than in cohort 1 (58.7% vs. 34.4%, *P* = 0.0008).

Apart from the different proportions of pediatric cases and the number of glomeruli, the 2 cohorts exhibited no relevant differences, which justified the subsequent pooled analysis. In the overall cohort of 194 biopsies, endocapillary hypercellularity was the most frequently observed active lesion (72.7%), followed by neutrophils/karyorrhexis (58.3%). Fibrinoid necrosis was the least commonly reported lesion (10.8%), whereas hyaline deposits and interstitial inflammation were found in 27.3% and 37.6% of the kidney biopsies, respectively. Interestingly, hyaline deposits were never observed in absence of endocapillary hypercellularity and in most cases there were neutrophils/karyorrhexis.

Among the chronic lesions, total glomerulosclerosis stood out, being present in 56.7% of the biopsies. Tubular atrophy (46.4%) and interstitial fibrosis (48.5%) were the next most frequently observed lesions and were almost always found together. Fibrous crescents were encountered only in 18.6% of the cases.

**Figure 4:**
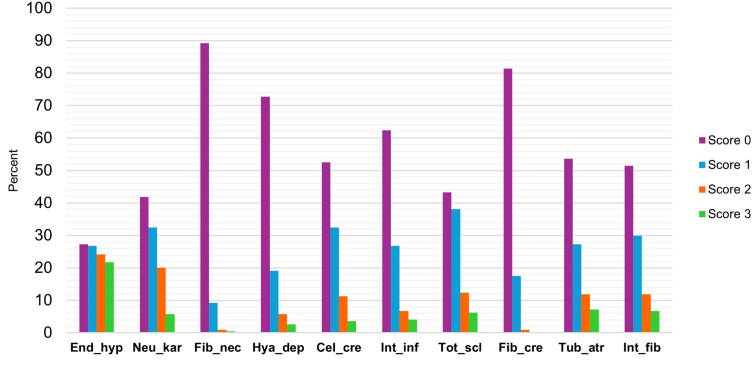
Score distribution of the active and chronic lesions in the 194 kidney biopsies. Cel_cre, cellular/fibrocellular crescents; End_hyp, endocapillary hypercellularity; Fib_cre, fibrous crescents; Fib_nec, fibrinoid necrosis; Hya_dep, hyaline deposits; Int_fib, interstitial fibrosis; Int_inf, interstitial inflammation; Neu_kar, neutrophils/karyorrhexis; Tot_scl, total glomerulosclerosis score; Tub_atr, tubular atrophy.

The bar chart shows the distribution of active and chronic lesion scores in all 194 biopsies (Figure 4). Endocapillary hypercellularity displayed an almost perfect distribution of scores from 0 to 3, with approximately 25% of biopsies corresponding to each score. The other lesions displayed a more uneven score distribution, because most biopsies had a score of either 0 or 1. Fibrous crescents never reached the highest score. Interestingly, fibrinoid necrosis, which is one of the lesions with a doubled score in the NIH AI, was very rare, and only 3 patients had scores of 2 or 3. This means that 98.5% of all patients did not have this lesion at all or had only in < 25% of their analyzed glomeruli. Combining the doubled scores for fibrinoid necrosis and cellular crescents, revealed that only 1 patient reached a total score of 10, whereas 2 patients had a total score of 8.

### Correlation Analysis

The similarities in NIH scores, as well as in active and chronic lesion distribution, justified the combination of the 2 cohorts in further correlation analysis. Nonparametric Spearman coefficients and parametric Pearson coefficients were almost identical. In view of the factor analysis which is based on a linear parametric model, Pearson coefficients are reported (Supplementary Table S3).

Among the active lesions, the strongest correlation was observed between endocapillary hypercellularity and neutrophils/karyorrhexis (*r* = 0.78, *P* < 0.0001). Both lesions were modestly associated with hyaline deposits, cellular crescents, and interstitial inflammation (*P* < 0.0001). Fibrinoid necrosis demonstrated a weak correlation with cellular crescents (*r* = 0.23, *P* = 0.0015) and neutrophils/karyorrhexis (*r* = 0.20, *P* = 0.0045).

Among the chronic lesions, there was a strong correlation between tubular atrophy and interstitial fibrosis (*r* = 0.98, *P* < 0.0001), which almost always displayed the same score, but also with total glomerulosclerosis (*r* = 0.60 and *r* = 0.61, *P* < 0.0001). In addition, tubular atrophy and interstitial fibrosis demonstrated a weak correlation with interstitial inflammation (*r* = 0.15, *P* = 0.034 and *P* = 0.039). Despite being chronic lesions, fibrous crescents exhibited the highest correlation with cellular crescents (*r* = 0.33, *P* < 0.0001), as well as a weak correlation with endocapillary hypercellularity (*r* = 0.17, *P* = 0.015), neutrophils/karyorrhexis (*r* = 0.16, *P* = 0.027), interstitial inflammation (*r* = 0.17, *P* = 0.018) and total glomerulosclerosis (*r* = 0.17, *P* = 0.019).

### Factor Analysis

To better understand the underlying factors that explain the correlations and variation among the observed variables, an exploratory factor analysis was conducted. Looking at the eigenvalues of the correlation matrix, which give an indication of how many factors are needed to deliver a good explanation of the data and on the amount of variance explained by each factor (Table 2), we observed that the first 2 eigenvalues were quite dominant (2.79 and 2.41), together explaining 52.0% of the total variation in the data. However, according to the Kaiser criterion, which suggests considering at least as many factors as there are eigenvalues > 1, a minimum of a third factor should be considered. In contrats, eigenvalues 4 to 5 were also close to 1 and, only after eigenvalue 5, was there a greater gap in the magnitude of eigenvalues; with 5 factors, a major fraction of the total variation (82.5%) was explained. Therefore, all solutions with 2 to 5 factors appeared to be reasonable and were investigated in detail.

**Table 2:** Eigenvalues of the correlation matrix

Factor	Eigenvalue	Difference to predecessor	Proportion (%)	Cumulative proportion (%)
1	2.79		27.9	27.9
2	2.41	0.38	24.1	52.0
3	1.28	1.13	12.8	64.8
4	0.95	0.32	9.5	74.3
5	0.83	0.13	8.3	82.5
6	0.59	0.24	5.9	88.4
7	0.50	0.09	5.0	93.4
8	0.43	0.07	4.3	97.7
9	0.21	0.22	2.1	99.8
10	0.02	0.18	0.2	100

The factor patterns in the 2-factors solution (obtained after Varimax rotation to achieve optimal separation) are displayed in Table 3. Factor 1 had high loadings in variables reflecting the chronic lesions, especially tubular atrophy, interstitial fibrosis, and total glomerulosclerosis, whereas factor 2 had high loadings in the variables reflecting the active lesions. In particular, 85% and 86% of the variability in endocapillary hypercellularity and neutrophils, respectively, were represented by factor 2. Fibrinoid necrosis was not well-represented by factor 2. Notably, fibrous crescents were moderately represented by both factors.

**Table 3:** Factor loadings (%) in the 2-factor solution after VARIMAX rotation

Variables	Factor 1	Factor 2
End_hyp	−2	85^a^
Neu_kar	−6	86^a^
Fib_nec	−2	24
Hya_dep	−2	45
Cell_cre	4	73^a^
Int_inf	20	52^a^
Tot_scl	79^a^	−4
Fib_cre	26	36
Tub_atr	95^a^	3
Int_fib	95^a^	5

Printed values are multiplied by 100 and rounded to the nearest integer. Values > 0.51 are flagged by an “^a^”.

Interestingly, looking at the correlation coefficients between factor 1, factor 2, and the NIH AI and CI scores, we found a strong correlation between factor 1 and the NIH CI score (*r* = 0.99, *P* < 0.0001) and between factor 2 and the NIH AI score (*r* = 0.98, *P* < 0.0001). In summary, the solution with 2 factors is very similar to the established NIH indices. The weighting of individual lesions is somewhat different; specifically, doubling the scores for fibrinoid necrosis and cellular crescent does not seem to be justified. Furthermore, the inclusion of fibrous crescents into the NIH CI and the addition of fibrinoid necrosis to the NIH AI appear to be questionable.

Increasing the number of factors considered in the analysis allowed for the explanation of a progressively higher percentage of the variability in the data (≤ 83% with 5 factors) (Supplementary Table S4). However, whereas factor 1 remained consistently characterized by total glomerulosclerosis, interstitial fibrosis, and tubular atrophy, collectively reflecting the NIH CI, active lesions tended to reorganize as the number of factors increased and they correlated progressively less strongly with the NIH AI.

## Discussion

In this histopathological study, we have for the first time described the performance of NIH AI and CI scores, by examining kidney biopsy findings from 2 distinct cohorts of adult and pediatric patients with LN. We observed similar results across the 2 cohorts, with respect to both the distribution of the NIH AI and NIH CI scores and the histopathological lesions.

A first key point to emphasize is that, when considering all classes of the LN classification system together, there can be low scores (even a score of 0) in the NIH AI; however, the maximum score never exceeds 16. This means that the upper range of possible scores from 17 to 24 is never reached, which has important implications for the interpretation of the AI score. Most likely, a value of 16 of 24 would be interpreted by pathologists and clinicians as a “moderate” score, indicating that 2 of 3 of the maximum activity value is reached. Our study shows that across 194 biopsies, a score of 16 actually represents the maximum amount of activity that can be expected in LN. Although this observation can be observed from the results of other studies, its significance was previously neglected and its meaning was left unquestioned.^14^^,^^17^^,^^20^, ^21^, ^22^, ^23^ Notably, the limitation on attainable NIH AI scores does not stem from individual subcategories failing to reach their maximum potential; interestingly, all lesions exhibited the full scoring range of 0 to 3. It is the low probability of all glomeruli within a biopsy reaching maximum scores for every lesion which accounts for the effect of the maximum score of 16.^20^^,^^22^

The NIH CI behaves differently from the NIH AI, showing a more even distribution of the scores. Many biopsies received a score of 0, the majority had scores between 1 and 4; however, there were biopsies reaching a score of up to 10, which is quite close to highest that can be reached, namely 12. Nevertheless, when comparing the NIH AI and CI, there is an important caveat to consider: the NIH AI consists of 6 subcategories, with 2 of them weighted more heavily, effectively treating the score as if it had eight subcategories from a statistical perspective; whereas the NIH CI has only 4 subcategories. Given the overall low likelihood of achieving the maximum score across all subcategories, for the NIH AI, there is certainly a wider range of total scores that are seldom reached. Moreover, whereas the NIH CI has a fairly compact number of lesions which seem to cover practically the whole domain of chronicity in LN, the NIH AI likely overlooks several potentially meaningful lesions such as thrombotic microangiopathy, lupus vasculitis and lupus podocytopathy.^24^, ^25^, ^26^, ^27^

Another key aspect is the strong association between the NIH indices and the ISN/RPS histological classes, with classes III, IV, and mixed exhibiting higher scores compared with the others. This is the clear consequence of how these indices were built. Whereas nearly all chronic lesions (except for fibrous crescents) can be found across classes I, II, and V, in the case of the NIH AI; 5 out of 6 lesions are, by definition, only present in classes III and IV. Consistently, classes I, II, and V mostly showed a NIH AI score of 0 in our cohorts. By definition, these classes will never have a score > 3 (only scores for interstitial inflammation would not move the biopsy into another class), raising the question of whether the NIH AI is truly useful for biopsies of those classes.

This study compared the NIH AI and CI scores between adult and pediatric patients, although the latter accounted for only a small proportion of the whole cohort. The absence of a significant difference in NIH AI scores, combined with significantly higher NIH CI scores in adults, suggests that, despite comparable levels of active inflammation, adults accumulate more chronic renal damage than children. Potential explanations for this include longer disease duration in adults, providing additional time for chronicity to develop, the potential impact of comorbidities in exacerbating damage, and varying treatment responses between pediatric and adult patients.

Through an exploratory factor analysis, which is an unbiased, purely mathematical process, we investigated how the activity and chronicity variables combine within LN biopsies, independently of the NIH AI and CI. Remarkably, we observed that 2 main underlying factors, factor 1 and factor 2, explained 52% of the variation in the data, closely reflecting the NIH CI and AI, respectively. Moreover, 85% and 86% of the variability in endocapillary hypercellularity and neutrophils/karyorrhexis, respectively, were represented by factor 2. Cellular crescents and interstitial inflammation were moderately represented by factor 2. Hyaline deposits were not strongly defined by factor 2; however, their loadings increased in factor 3 to 5 analyses, consistently grouping them with endocapillary hypercellularity and neutrophils/karyorrhexis. Although a recent study demonstrated that hyaline deposits were significantly associated with higher proteinuria and lower serum albumin levels, showing the strongest correlation with clinical activity,^21^ more robust evidence is required to confirm whether these deposits offer additional value in assessing disease severity or rather contribute redundant information in relation to other histological lesions.

Among chronic lesions, total glomerulosclerosis, tubular atrophy, and interstitial fibrosis exhibited a strong correlation with each other and, independently from the number of factors considered in the analysis, consistently grouped together in explaining the main variability of factor 1. Fibrous crescents behaved differently, showing only a slight correlation with total glomerulosclerosis and the strongest correlation with cellular/fibrocellular crescents. Consistently, fibrous crescents were moderately represented by both factor 1 and factor 2. Increasing the number of factors to 5, fibrous crescents tended to deviate from both chronic and active lesions. This suggests that fibrous crescents might still retain some degree of activity and potentially represent a continuum between active and chronic damage. This finding aligns with the study by Fava *et al.*,^28^ where urine proteomic signatures revealed that fibrous crescents exhibit characteristics more akin to activity-related lesions despite being traditionally classified as inactive lesions and included in the NIH CI.

Factor analysis provided useful insights into the behavior of the variables included in the NIH AI and CI and will certainly be useful in guiding future studies aimed at modifying these indices. To optimize the utility of the NIH AI and CI, it would be important to examine how modifying the subcategories might impact the indices. There are a few additional chronic lesions in LN that could be considered for inclusion in a new CI (e.g., adhesions, arterial intimal fibrosis). In contrast, it remains to be ascertained whether reducing the number of subcategories would maintain its overall effectiveness. This is particularly relevant given the strong correlations observed between certain lesions, such as tubular atrophy and interstitial fibrosis, which could potentially be combined to streamline NIH CI’s application. The discussion surrounding NIH AI is considerably more complex, beginning with the scoring of individual subcategories. Austin *et al.*^3^ assigned double weight to cellular/fibrocellular crescents and fibrinoid necrosis because of their established association with more severe and rapidly progressive disease in previous studies. However, whereas cellular crescents are frequently observed, fibrinoid necrosis seems to have a very low prevalence in LN biopsies. Consequently, its doubled score might disproportionately influence the overall NIH AI score without accurately reflecting the true disease activity or prognosis in these patients. Another crucial aspect is that LN encompasses numerous active lesions beyond those currently incorporated into the NIH AI score. The effects of including additional lesions, whether through modifications to the existing scoring system or selective omission of certain current components, remain to be explored.

Our study has several limitations. First, the decision-making process regarding the indication and timing of renal biopsies is likely to have varied between centers, introducing potential selection bias. Second, biopsies were obtained at different points in the disease course (diagnosis and relapse), which inevitably captures different stages of disease activity and chronicity. Furthermore, immunosuppressive treatment administered before the biopsy could have influenced the observed histological features, potentially masking or modifying the true extent of renal damage in the acute phase of disease. Lastly, though the analysis of biopsies by a single pathologist ensured data uniformity, it precluded a verification of whether interobserver variability persists following the 2018 classification update, which has demonstrated a superior correlation with renal prognosis.^16^

By encompassing a broad spectrum of LN biopsies, which represent diverse clinical scenarios, this study provides a comprehensive understanding of how the NIH AI and CI perform across the full range of clinical presentations, enabling a more precise assessment of their strengths and weaknesses in real-world clinical practice. Hopefully, the findings of this study will pave the way for a careful and effective modification of the current scoring system.

## Disclosure

DD has received grants from the medical Research Council and the National Institute for Health and Care Research, outside the submitted work; he has also received consultation fees and payments for presentations and participation on Advisory Boards from GSK, UCB, and Vifor CSL. IB has received grants from Novartis and consultation fees from Aurina, Novartis, Hansa, and Alentis, outside the submitted work; she is also the Director of BiPath. All the other authors declared no competing interests.

## Supplementary Material

Supplementary File (PDF) **Table S1.** Stratified analyses to compare both cohorts within adults and pediatric patients and within patients with ≤ 16 glomeruli and with ≥ 17 glomeruli (median split). **Table S2.** Demographic and histopathological data of cohort 2 patients, stratified by ethnicity. **Table S3.** Correlations between active and chronic lesions in the 194 kidney biopsies. Significant correlations (*P* < 0.05) are in bold. **Table S4.** Factor loadings given in percentages. The cumulative percentage of explained variability by adopting an increasing number of factors (1–5) is reported. By increasing the number of factors, chronic lesions (interstitial fibrosis, tubular atrophy and total glomerulosclerosis) tend to retain high loadings in factor 1, while active lesions reorganize into smaller groups. STROBE Checklist.
