# Supplementary material for: Characterizing the NIH Activity and Chronicity Indices in 2 Independent Lupus Nephritis Cohorts
Source: Kidney Int Rep. 2025 Nov 20;11(2):103686. doi: 10.1016/j.ekir.2025.11.011 (PMC12800592; doi:10.1016/j.ekir.2025.11.011)
Supplement: Supplementary File (PDF) — Table S1. Stratified analyses to compare both cohorts within adults and pediatric patients and within patients with ≤ 16 glomeruli and with ≥ 17 glomeruli (median split). Table S2. Demographic and histopathological data of cohort 2 patients, stratified by ethnicity. Table S3. Correlations between active and chronic lesions in the 194 kidney biopsies. Significant correlations (P < 0.05) are in bold. Table S4. Factor loadings given in percentages. The cumulative percentage of explained variability by adopting an increasing number of factors (1–5) is reported. By increasing the number of factors, chronic lesions (interstitial fibrosis, tubular atrophy and total glomerulosclerosis) tend to retain high loadings in factor 1, while active lesions reorganize into smaller groups. STROBE Checklist. [file mmc1.pdf]

Supplementary Material

Supplementary Table S1

Supplementary Table S2

Supplementary Table S3

Supplementary Table S4

	NIH AI	NIH CI
**Pediatric patients**	p=0.84	p=0.52
**Adult patients**	p=.010	p=0.15
**< 17 glomeruli**	p=0.81	p=0.21
**≥ 17 glomeruli**	P=0.34	p=0.92

Supplementary Table S1. Stratified analyses to compare both cohorts within adults and paediatric patients and within patients with ≤16 glomeruli and with ≥ 17 glomeruli (median split).

	Caucasian (n=40; 38.5%)	Afro-Caribbean (n=39; 37.5%)	Asian (n=21; 20.2%)	Other (n=4; 3.8%)	Cohort 2 (n=104)	p-value
**Age, *median (Q1-Q3)***	32 (27-39)	31 (22-41)	30 (22-34)	33 (32-34)	32 (25-40)	0.45
**< 18 years, *n (%)***	3 (7.5)	5 (12.8)	4 (19.0)	-	12 (11.5)	0.50
**Female gender, *n (%)***	32 (80.0)	34 (87.2)	15 (71.4)	4 (100)	85 (81.7)	0.36
**N° of glom, *median (Q1-Q3)***	21 (13-30)	19 (12-29)	23 (17-34)	19 (13-26)	21 (13-29)	0.34
**Class I, *n (%)***	1 (2.5)	1 (2.6)	-	-	2 (1.9)	
**Class II, *n (%)***	4 (10.0)	6 (15.4)	1 (11.1)	1 (25.0)	12 (11.5)	
**Class III, *n (%)***	11 (27.5)	13 (33.3)	4 (19.0)		28 (26.9)	
**Class IV, *n (%)***	20 (50.0)	14 (35.9)	12 (57.1)	3 (75.0)	49 (47.1)	0.93
**Class V, *n (%)***	2 (5.0)	3 (7.7)	3 (14.3)	-	8 (7.7)	
**Class III+V, *n (%)***	1 (2.5)	1 (2.6)	-	-	2 (1.9)	
**Class IV+V, *n (%)***	1 (2.5)	1 (2.6)	1 (4.8)	-	3 (2.9)	
**Classes III, IV and mixed, *n (%)***	33 (82.5)	29 (74.4)	17 (81.0)	3 (75.0)	82 (78.8)	0.83
**NIH AI score, *median (Q1-Q3)***	4 (1-8)	4 (1-7)	7 (2-10)	8 (4-10)	4 (1-8)	0.33
**Endocapillary hypercellularity, *n (%)***	31 (77.5)	25 (64.1)	15 (71.4)	3 (75.0)	74 (71.2)	0.62
**Neutrophils/karyorrhexis, *n (%)***	22 (55.0)	21 (53.8)	14 (66.7)	3 (75.0)	60 (57.7)	0.67
**Fibrinoid necrosis, *n (%)***	3 (7.5)	1 (2.6)	5 (23.8)	-	9 (8.7)	**0.039**
**Hyaline deposits, *n (%)***	11 (27.5)	4 (10.3)	9 (42.9)	2 (50.0)	26 (25.0)	**0.023**
**Cellular/fibrocellular crescents, *n (%)***	21 (52.5)	23 (59.0)	14 (66.7)	1 (25.0)	61 (58.7)	0.65
**Interstitial inflammation, *n (%)***	15 (37.5)	16 (41.0)	10 (47.6)	3 (75.0)	44 (42.3)	0.49
**NIH CI score, *median (Q1-Q3)***	2 (0-4)	1 (0-5)	1 (0-4)	1 (1-2)	1 (0-4)	0.81
**Total glomerulosclerosis, *n (%)***	23 (57.5)	24 (61.5)	10 (47.6)	4 (100)	61 (58.7)	0.25
**Fibrous crescents, *n (%)***	11 (27.5)	7 (17.9)	5 (23.8)	-	23 (22.1)	0.52
**Tubular atrophy, *n (%)***	20 (50.0)	16 (41.0)	9 (42.9)	1 (25.0)	46 (44.2)	0.72
**Interstitial fibrosis, *n (%)***	21 (52.5)	16 (41.0)	9 (42.9)	1 (25.0)	47 (45.2)	0.61

Supplementary Table S2. Demographic and histopathological data of cohort 2 patients, stratified by ethnicity.

Pearson Correlation Coefficients, N=194<br>Prob > |r| under H0: Rho=0										
	**End_hyp**	**Neu_kar**	**Fib_nec**	**Hya_dep**	**Cel_cre**	**Int_inf**	**Tot_scl**	**Fib_cre**	**Tub_atr**	**Int_fib**
**End_hyp**	1	**0.78<br><.0001**	0.07<br>0.34	**0.42<br><.0001**	**0.44<br><.0001**	**0.28<br><.0001**	-0.05<br>0.53	**0.17<br>0.015**	0.05<br>0.52	0.05<br>0.46
	**Neu_kar**	1	**0.20<br>0.0045**	**0.35<br><.0001**	**0.46<br><.0001**	**0.28<br><.0001**	-0.08<br>0.26	**0.16<br>0.027**	0.02<br>0.77	0.03<br>0.68
		**Fib_nec**	1	-0.11<br>0.10	**0.23<br>0.0015**	0.05<br>0.53	0.04<br>0.60	0.06<br>0.38	-0.03<br>0.68	-0.02<br>0.78
			**Hya_dep**	1	0.10<br>0.17	0.07<br>0.31	-0.04<br>0.62	0.01<br>0.90	0.03<br>0.57	0.05<br>0.48
				**Cel_cre**	1	**0.43<br><.0001**	0.04<br>0.57	**0.33<br><.0001**	0.01<br>0.93	0.03<br>0.73
					**Int_inf**	1	0.10<br>0.18	**0.17<br>0.018**	**0.15<br>0.034**	**0.15<br>0.039**
						**Tot_scl**	1	**0.17<br>0.019**	**0.60<br><.0001**	**0.61<br><.0001**
							**Fib_cre**	1	0.15<br>0.038	0.16<br>0.025
								**Tub_atr**	1	**0.98<br><.0001**
									**Int_fib**	1

Supplementary Table S3. Correlations between active and chronic lesions in the 194 kidney biopsies. Significant correlations (p<.05) are in bold.

Legend: End_hyp: Endocapillary hypercellularity, Neu_kar: Neutrophils/karyorrhexis, Fib_nec: Fibrinoid necrosis, Hya_dep: Hyaline deposits, Cel_cre: Cellular/Fibrocellular crescents, Int_inf: Interstitial inflammation, Tot_scl: Total glomerulosclerosis score, Fib_cre: Fibrous crescents, Tub_atr: Tubular atrophy, Int_fib: Interstitial fibrosis.

Factor loadings (%)	Cumulative percentage of explained variability														
	52%		65%			74%				83%					
	F1	F2	F1	F2	F3	F1	F2	F3	F4	F1	F2	F3	F4	F5
**End_hyp**	-2	**85**	-2	**84**	30	-1	**8**	29	9	-1	**85**	25	9	12
**Neu_kar**	-6	**86**	-6	**77**	40	-4	**81**	28	27	-3	**81**	27	27	9
**Fib_nec**	-2	24	-10	-17	**61**	1	2	2	**95**	1	3	2	**95**	1
**Hya_dep**	-2	45	4	**77**	-27	4	**75**	-12	-29	4	**76**	-13	-29	-6
**Cell_cre**	4	**73**	-3	35	**74**	-5	31	**72**	27	-4	31	**63**	27	37
**Int_inf**	20	**52**	15	27	**52**	9	18	**69**	-4	12	10	**92**	-7	-1
**Tot_scl**	**79**	-4	**78**	-12	12	**78**	-12	11	6	**78**	-10	2	6	13
**Fib_cre**	26	36	20	5	**54**	14	-5	**71**	-3	13	6	9	0	**96**
**Tub_atr**	**95**	3	**96**	6	2	**96**	6	6	-4	**96**	5	5	-4	0
**Int_fib**	**95**	5	**96**	7	3	**96**	7	6	-3	**96**	6	5	-3	2

Supplementary Table S4. Factor loadings given in percentages. The cumulative percentage of explained variability by adopting an increasing number of factors (from 1 to 5) is reported. By increasing the number of factors, chronic lesions (interstitial fibrosis, tubular atrophy and total glomerulosclerosis) tend to retain high loadings in factor 1, while active lesions reorganize into smaller groups.

Legend: End_hyp: Endocapillary hypercellularity, Neu_kar: Neutrophils/karyorrhexis, Fib_nec: Fibrinoid necrosis, Hya_dep: Hyaline deposits, Cel_cre: Cellular/Fibrocellular crescents, Int_inf: Interstitial inflammation, Tot_scl: Total glomerulosclerosis score, Fib_cre: Fibrous crescents, Tub_atr: Tubular atrophy, Int_fib: Interstitial fibrosis.

STROBE Statement—Checklist of items that should be included in reports of ***cohort studies***

	Item No	Recommendation	Page No
**Title and abstract**	1	(*a*) Indicate the study's design with a commonly used term in the title or the abstract	1,3
		(*b*) Provide in the abstract an informative and balanced summary of what was done and what was found	3
**Introduction**			
Background/rationale	2	Explain the scientific background and rationale for the investigation being reported	5,6
Objectives	3	State specific objectives, including any prespecified hypotheses	6
**Methods**			
Study design	4	Present key elements of study design early in the paper	6-9
Setting	5	Describe the setting, locations, and relevant dates, including periods of recruitment, exposure, follow-up, and data collection	6,7
Participants	6	(*a*) Give the eligibility criteria, and the sources and methods of selection of participants. Describe methods of follow-up	6,7
		(*b*) For matched studies, give matching criteria and number of exposed and unexposed	NA
Variables	7	Clearly define all outcomes, exposures, predictors, potential confounders, and effect modifiers. Give diagnostic criteria, if applicable	6,7
Data sources/ measurement	8*	For each variable of interest, give sources of data and details of methods of assessment (measurement). Describe comparability of assessment methods if there is more than one group	6,7
Bias	9	Describe any efforts to address potential sources of bias	8,9
Study size	10	Explain how the study size was arrived at	6,7
Quantitative variables	11	Explain how quantitative variables were handled in the analyses. If applicable, describe which groupings were chosen and why	8
Statistical methods	12	(*a*) Describe all statistical methods, including those used to control for confounding	8,9
		(*b*) Describe any methods used to examine subgroups and interactions	8-9
		(*c*) Explain how missing data were addressed	NA
		(*d*) If applicable, explain how loss to follow-up was addressed	NA
		(*e*) Describe any sensitivity analyses	8
**Results**			
Participants	13*	(a) Report numbers of individuals at each stage of study—eg numbers potentially eligible, examined for eligibility, confirmed eligible, included in the study, completing follow-up, and analysed	6,7
		(b) Give reasons for non-participation at each stage	NA
		(c) Consider use of a flow diagram	NA
Descriptive data	14*	(a) Give characteristics of study participants (eg demographic, clinical, social) and information on exposures and potential confounders	9, 10
		(b) Indicate number of participants with missing data for each variable of interest	9,10
		(c) Summarise follow-up time (eg, average and total amount)	NA
Outcome data	15*	Report numbers of outcome events or summary measures over time	9-15

Main results	16	(*a*) Give unadjusted estimates and, if applicable, confounder-adjusted estimates and their precision (eg, 95% confidence interval). Make clear which confounders were adjusted for and why they were included<br>(*b*) Report category boundaries when continuous variables were categorized<br>(*c*) If relevant, consider translating estimates of relative risk into absolute risk for a meaningful time period	9-15<br><br>9,10<br>NA
Other analyses	17	Report other analyses done—eg analyses of subgroups and interactions, and sensitivity analyses	12-15
**Discussion**			
Key results	18	Summarise key results with reference to study objectives	15-18
Limitations	19	Discuss limitations of the study, taking into account sources of potential bias or imprecision. Discuss both direction and magnitude of any potential bias	19-20
Interpretation	20	Give a cautious overall interpretation of results considering objectives, limitations, multiplicity of analyses, results from similar studies, and other relevant evidence	15-20
Generalisability	21	Discuss the generalisability (external validity) of the study results	18-20
**Other information**			
Funding	22	Give the source of funding and the role of the funders for the present study and, if applicable, for the original study on which the present article is based	21

*Give information separately for exposed and unexposed groups.

Note: An Explanation and Elaboration article discusses each checklist item and gives methodological background and published examples of transparent reporting. The STROBE checklist is best used in conjunction with this article (freely available on the Web sites of PLoS Medicine at http://www.plosmedicine.org/, Annals of Internal Medicine at http://www.annals.org/, and Epidemiology at http://www.epidem.com/). Information on the STROBE Initiative is available at http://www.strobe-statement.org.
